# Researchers’ perspectives on methodological challenges and outcomes selection in interventional studies targeting medication adherence in rheumatic diseases: an OMERACT-adherence study

**DOI:** 10.1186/s41927-021-00193-4

**Published:** 2021-07-08

**Authors:** Shahrzad Salmasi, Ayano Kelly, Susan J. Bartlett, Maarten de Wit, Lyn March, Allison Tong, Peter Tugwell, Kathleen Tymms, Suzanne Verstappen, Mary A. De Vera, On behalf of the OMERACT-Adherence working group

**Affiliations:** 1 Collaboration for Outcomes Research and Evaluation, Faculty of Pharmaceutical Sciences, University of British Columbia, 2405 Wesbrook Mall, Vancouver, BC V6T 1Z3 Canada; 2 Arthritis Research Canada, Richmond, Canada; 3 College of Health and Medicine, Australian National University, Canberra, ACT Australia; 4 Canberra Rheumatology, Canberra, ACT Australia; 5 Centre for Kidney Research, The Children’s Hospital at Westmead, Sydney, NSW Australia; 6 Department of Medicine, McGill University and Research Institute, McGill University Health Centres, Montreal, Canada; 7 Division of Rheumatology, Johns Hopkins School of Medicine, Baltimore, MD USA; 8 OMERACT Patient Research Partner, Amsterdam, Netherlands; 9 Institute of Bone and Joint Research, Kolling Institute of Medical Research, Sydney, NSW Australia; 10 Department of Rheumatology, Royal North Shore Hospital, Sydney, NSW Australia; 11 Northern Clinical School, The University of Sydney, Sydney, NSW Australia; 12 Sydney School of Public Health, The University of Sydney, Sydney, NSW Australia; 13 Department of Medicine, University of Ottawa, Ottawa, Ontario Canada; 14 Department of Rheumatology, Canberra Hospital, Canberra, ACT Australia; 15 Centre for Epidemiology Versus Arthritis, Centre for Musculoskeletal Research, Faculty of Biology, Medicine and Health, University of Manchester, Manchester Academic Health Science Centre, Manchester, UK; 16 NIHR Manchester Biomedical Research Centre, Manchester University Hospitals NHS Foundation Trust, Manchester Academic Health Science Centre, Manchester, UK

**Keywords:** Rheumatology, Medication adherence, Qualitative research

## Abstract

**Background:**

Research on adherence interventions in rheumatology is limited by methodological issues, particularly heterogeneous outcomes. We aimed to describe researchers’ experiences with conducting interventional studies targeting medication adherence in rheumatology and their perspectives on establishing core outcomes.

**Methods:**

Semi-structured interviews using audio conference were conducted with researchers who had conducted an adherence study of any design in the past 10 years. Data collection and thematic analysis were performed iteratively, until saturation.

**Results:**

We interviewed 13 researchers, most of whom worked in academia and specialized in epidemiology and/or health services research. We identified three themes: 1) improving measurement of adherence (considering all phases of adherence, using appropriate and relevant measures, and establishing clinically meaningful thresholds); 2) challenges in designing and appraising adherence intervention studies (considering the confusion over a plethora of outcomes, difficulties with powering studies to demonstrate meaningful changes, and suboptimal descriptions of adherence interventions in published studies); and 3) advancing outcome assessment in adherence intervention studies (capturing rationale for developing a core domain set as well as recommendations and anticipated challenges by participants).

**Conclusions:**

Uniquely gathering perspectives from international adherence researchers, our findings led to researcher-informed recommendations for improving adherence research including specifying the targeted adherence phase in designing interventions and studies and providing a glossary of terms to promote consistency in reporting. We also identified recommendations for developing a core domain set for interventional studies targeting medication adherence including involvement of patients, clinicians, and other stakeholders and methodological and practical considerations to establish rigor and support uptake.

**Supplementary Information:**

The online version contains supplementary material available at 10.1186/s41927-021-00193-4.

## Background

Medication non-adherence in rheumatic diseases - as high as 90% in gout, 70% in rheumatoid arthritis (RA), and 43% in systemic lupus erythematosus (SLE) [1, 2] - has been associated with adverse outcomes including morbidity (in gout [3], RA [4], SLE [5]) and increased health care utilization and cost (in gout [3], RA [4], SLE [6]). There have been attempts to develop adherence interventions to support medication taking among patients with rheumatic diseases [7]. However, a 2019 systematic review assessing the scope of outcomes in interventional studies targeting medication adherence in rheumatology showed substantial heterogeneity with 71 outcome domains identified among 53 included studies [8]. Such heterogeneity and lack of standard approaches to outcomes selection and evaluation preclude meaningful comparisons across studies, hampering efforts to address medication adherence in rheumatology. In response to these identified gaps, the Outcome Measures in Rheumatology (OMERACT) Adherence Working Group initiated the development of a core domain set for interventional studies of medication adherence among patients with rheumatic diseases [9]. We applied the OMERACT definition of a core domain set as the ‘minimum set of outcome domains that should be measured and reported in every clinical trial.’ [10] Gathering input from stakeholders is essential to this five-phase effort [10]. As the third phase of planned studies, our objective was to describe researchers’ experiences with conducting interventional studies targeting medication adherence in rheumatology and their perspectives on establishing a core domain set for such studies.

## Methods

### Study design

We conducted an international qualitative study as part of the OMERACT-Adherence Working Group.

### Participants

Researchers were eligible if they were English-speaking and had conducted an interventional study targeting medication adherence in the past 10 years using any design (e.g. clinical trials, observational studies). We drew from our collegial and professional networks internationally to identify potential participants. We utilized purposive sampling [11] to collect a broad range of perspectives and maximize variation with respect to demographic characteristics, research discipline (e.g. epidemiology, psychology), and methodological expertise. Potential participants were approached with an email invitation from the study coordinator containing information about the study objectives and processes, and a link to our online consent form. After providing written informed consent, participant’s demographic information were collected online through Qualtrics®.

### Data collection

The first author (SS), a PhD candidate, who is a pharmacist with a graduate degree in qualitative research, conducted the semi-structured interviews through audio conference from August 2019 to January 2020, without the presence of non-participants. The interviewer had no prior relationship with the participants. The interview guide (Additional file 1) was developed by Working Group members and pilot tested with one participant to determine question clarity, content validity, and average time required for completion. Participants were given the opportunity to express additional views at the end of their interview. Each interview was recorded to allow the interviewer to focus on the verbal prompts. Field notes were taken as needed. Interviews were transcribed verbatim by a professional transcriber.

### Analysis

Data was analyzed using inductive thematic analysis following the steps recommended by Braun and colleagues [12]. After immersing in the data by repeatedly reading interview transcripts, the first author (SS) independently assigned as many different codes as relevant in a line-by-line approach using NVivo software® (QSR International Pty Ltd. Version 11) [13]. Homogeneity and heterogeneity between the codes were then assessed to construct categories and eventual themes [12]. Data collection and analysis were conducted iteratively, with prior interviews informing subsequent ones, and until saturation was reached. Saturation was defined as the point where no new insights on constructed themes emerged, as discussed and confirmed by the first and senior authors (SS, MDV). Themes were further discussed among co-authors (AK, AT, MDV) and finalized. Results were shared with participants in a member-checking step to ensure accurate reflection of their shared perspectives. To increase the credibility of the study, representative participant quotes were provided to illustrate the results.

## Results

Altogether, 13 (5 females) researchers from seven countries (Australia, Belgium, Canada, Netherland, Thailand, United Kingdom, and United States of America) participated in the study (Table 1). All participants held a degree at least at the doctoral level and had led between two to five adherence research studies in the past five years (61.5%). Mean interview duration was 26 min. A majority worked in academia (75%) and specialized in epidemiology and/or health services research (61.5%).

**Table 1:** Participant characteristics (*N* = 13)

Characteristic	N (%)
**Sex**	
Male	8 (62%)
Female	5 (38%)
**Age (years)**	
31–40	4 (31%)
41–50	7 (54%)
51–60	1 (8%)
> 60	1 (8%)
**Number of adherence intervention studies involved in over the past 5 years as principal investigator**	
< 2	4 (31%)
2–5	8 (62%)
> 5	1 (8%)
**Number of adherence intervention studies involved in over the past 5 years as co-investigator**	
< 2	2 (15%)
2–5	5 (38%)
> 5	5 (38%)
Did not specify	1(8%)
**Highest education degree obtained**	
Post-doctorate	9 (69%)
Doctorate (e.g. PharmD, MBBS, PhD)	4 (31%)
**Research methodology expertise**^a^	
Randomized controlled trial	11 (85%)
Observational cohort	8 (62%)
Non-randomized controlled trial	7 (54%)
Qualitative	6 (46%)
Observational case-control	3 (23%)
Before-after interventional studies	2 (15%)
Other	2 (15%)
**Work setting**^a^	
Academia	9 (69%)
Hospital	5 (39%)
Clinic	2 (15%)
Government	2 (15%)
Industry	1 (77%)

^a^Participants could select more than one option. The percentages may therefore exceed 100%

We identified three themes: 1) improving measurement of adherence; 2) challenges in designing and appraising adherence intervention studies; and 3) advancing outcome assessment in adherence intervention studies. We describe each theme and corresponding categories in detail as follows and provide illustrative participant quotations in Table 2.

**Table 2:** Identified themes and representative participant quotes

Theme	Representative quote
**1. Improving measurement of adherence**	
**a. Considering all phases of adherence**	“… first of all it’s very important that researchers define exactly the impact on which element of medication adherence, this is very poorly defined typically if it’s improving initiation of treatment, implementation of treatment or persistence to treatment. Secondly choosing the appropriate measurement depending on the elements of adherence and providing the appropriate analysis.”
	“Long term the issue has been about measurements because people confuse and conflate various aspects of medication adherence. They talk just using the word adherence, not referring to precisely what it means […] there are three main phases which are initiation, start your first dose, there’s implementation which is what you do from one day to the next, and there’s persistence which is how long you continue on treatment before you give up. And most researchers to date have completely confused those three issues […] I think that’s hindered so much potential progress in adherence research.”
**b. Using appropriate and relevant adherence measures**	“Well adherence the big issue is that none of the outcomes are perfect. They all have their pros and cons either in terms of feasibility or their validity, sensitivity or specificity and it’s not clear to a researcher what combination of various outcomes should they use to capture that in the context of when they are measuring adherence to, within the study that they are, they are, that they are working on.”
	“I think the most challenging is how to measure the adherence because it’s so many ways to measure it but it’s not, not one is the gold standard, we don’t have a gold standard for adherence.”
**c. Establishing clinically meaningful thresholds**	“..often people use this kind of 80% cutoff and but then when you look at where that comes from […]. So it’s got very, just a relevance to a lot of the clinical condition(s), a lot of the diseases to which that cutoff’s applied. And it might well be that for some conditions and some medications and some patients maybe 60% adherence is fine”
	“I think one of the key areas is, is how much adherence you need for an individual to, to have a better result or a good result. I think there’s virtually no work on that that I’m familiar with and it’s very important. Why impose strict adherence criteria on people who may not need it, so in other words, can adherence be individualized?”
**2. Challenges in designing and appraising adherence intervention studies**	
**a. Confusion from a plethora of outcomes choices**	“I guess you have a kind of whole range of outcomes, so you can have psychological outcomes, say things like anxiety, depression, quality of life, self-reported, self-rated health, […] kind of perspectives on general wellbeing. There’s often measures of health care utilization and so things like attendance at hospital, attendance to primary care, nurse appointments and duration, things like times off work, or not able to undertake other kind of routine responsibilities, […]. And also I guess all the you know […] most relevant clinical outcomes.”
	“I would advocate using the EQ-5D quite often because it measures utility that can be used to estimate quality adjusted life years for calculating the cost-effectiveness of healthcare interventions, to assess the value for money of health technologies so I insist on including that, in my clinical trials as an outcome measure for economic analyses. But it depends on the trial. If it’s a trial of 20 patients and […] you just want to check to see if it (the intervention) has an impact on adherence then these would be very low sort of secondary outcomes on that scale. You could also have cost as an outcome […], but I’m interested in seeing whether that intervention represents good value for money.”
**b. Difficulty powering studies to demonstrate meaningful changes**	“I think one of the major things is often power because for, and kind of related to that is recruitment and in particular, because typically you have quite a large number of people in your sample that are already adherent […] and then recruiting sufficient numbers can be a challenge, particularly given that the people you often most want in your sample are the people who are non-adherent and often the people who are non-adherent are the people who are hardest to recruit.”
	“A key challenge though is power because you’d need an enormous study in many of these types of conditions in order to detect the (clinical) difference through improvements in adherence.”
**c. Suboptimal description of adherence interventions**	“… often the intervention contents are poorly recorded, […] there’s often no systemic use of key terms around the intervention contents so terms such as medication counselling or you know education are used to describe intervention contents and then often can mean lots and lots of different things, so it can be very hard to actually evaluate […] if you’ve got a trial where you’ve got nil results and it says we counselled patients on their medications, it’s very hard to conclude whether it had a nil result because medication counselling doesn’t work or if it’s a particular type of process that doesn’t work, or if often you know there’s all those issues around things like fidelity to protocols, […] so it might be that the intervention itself was beautifully designed but then it wasn’t delivered properly.”
**3. Advancing outcome assessment in adherence intervention studies**	
**a. Rationale for a core domain set for adherence intervention studies**	“… it will make trials more comparable and it will increase the likelihood that you’d be able to combine efforts internationally or you know with people doing kind of research or benefit work in different contexts. So I think that’s probably likely to increase the strength of the evidence base for what works and which I would hope would increase you know the willingness of policy makers to support services that used whatever the effect or the approach as identified were.”
	“I think it’s needed because if, if nothing it’ll give more clarity with regards to the measures, their limitation and how they apply to […] our conditions and will bring a lot of attention to, attention and emphasis to, to context and design sort of issues.”
**b. Prioritizing outcome for a core domain set for adherence intervention studies**	“it would be important to include some sort of patient centered outcome, you know some sort of objective measure of adherence ideally. We triangulate adherence ideally from a couple of different measurement perspectives so you know self-report we know has some real advantages in that we’re able to determine barriers or reasons for non-adherence but certainly patients on average tend to over-report their adherence.”
	“ultimately it’s the clinical outcome that relate to quality of life and survival that matter to the patient […] That hasn’t been done extensively. There are many examples in hypertension where they’ve assessed blood pressure perhaps as an intermediary clinical outcome but not necessarily anything more than that.”
	“what matters is the clinical. It may affect someone’s adherence and that’s very interesting but does it actually give them better control of their disease? It doesn’t matter if it changes their adherence, it only matters whether the patient has a good clinical outcome from it.”
	“Well it would depend on the design of the intervention so depending on how the intervention […] I would want to see those outcomes, those assessed and reported as outcomes. So for example, if it was an education intervention that’s designed to increase knowledge, I would want to see a measure of knowledge as one of the outcome measures or if it was an intervention that was designed to increase social support for medication taking, I would want to see a measure of you know perceptions of social support.”
**c. Challenges in developing and implementing a core domain set for adherence intervention studies**	“Context is also critically important, and I think generally not assessed well in the literature. There are often some practical limitations in data availability so if we’re doing things that are recruiting patients from the community, we might not have access to things like electronic health records or certainly certain countries don’t have access to electronic health record data so all of those things might impact how we would design outcome data collection.”
	“I think as a clinician my, the context for me is can I help to improve patients’ disease control. The context for a pharmaceutical company looking to improve adherence may be with a patient support program, it may be different, that might be about health economics for example and it also depends on what country and how the, what the economic model is for delivering healthcare in the country.”
	“The issue is very much to do with you know what’s the intention of the trial, is it a definitive study to show that so when an intervention let’s say has a demonstrable impact on health and outcomes or in the smaller scales do they to just check to see whether intervention has efficacy in improving adherence.”
	“I would just be clear on when and why you’re doing this, what the consequences and, and rewards are. And you know then if people chose not to do that that you know that’s, that’s something that they can do in an informed way.”
**d. Inclusivity and representativeness of a core domain set for stakeholders in adherence intervention research**	“… to get buy in so that always in these things the stakeholder involvement early on in making likely that it’s representative, which is very hard to do, but that’s I think being open and transparent about that and allowing it as a sort of an open source approach to a useful set of guidance if you like. I think getting the companies, regulators, patient groups, together to really support this and be enthusiastic’s a huge job.”
	“was thinking that still I believe in the core set but you can look at different points or different, with different glasses through that core set because sometimes as a researcher you have a glass looking at the effects of an intervention but also the mechanism, the […] effect whereas if you look at, from the patient point of view, you would like to know what means it for me as patients and they’re less interested in the mechanism behind it and clinicians tend to look mostly at the clinical aspects. So each of, of, of different point of view you have different demands from this core set.”

### 1. Improving measurement of adherence

This theme captured participants’ perspectives on the measurement of adherence, which is not just limited to interventional studies of medication adherence but also applies to all types of studies in the field, including descriptive studies that quantify the extent of adherence (or non-adherence) in a patient population and analytic studies that evaluate the association between adherence (or non-adherence) and outcome(s). Participants indicated limitations to the measurement of adherence and shared insights and recommendations for addressing them.

#### a. Considering the phases of adherence

Participants collectively called for consideration and better specification of the targeted phase of adherence in designing *both* interventions as well as studies to evaluate these. It was indicated by the majority that adherence research has been significantly hindered by confusion, conflation, and omission of the three phases of adherence (e.g. initiation, implementation of the dosing regimen, persistence with therapy). Participants provided examples - some studies only measure adherence for the period in which a patient is on therapy, with study follow-up terminating at the time when the drug is stopped, thus reflecting poor medication taking only at the implementation phase, while in other studies, patients are followed after therapy discontinuation, capturing poor medication taking during the implementation phase, as well as non-persistence with therapy. In citing prior works that have called for standardized definitions for phases of adherence, participants highlighted that efforts should go beyond use of terminologies but also in research practice with respect to explicit specification and measurement of these phases of adherence.

#### b. Using appropriate and relevant adherence measures

Participants indicated their preference for using multiple adherence measures in their studies, specifically a combination of objective measures which are not reliant on memory or affected by social desirability bias, combined with subjective measures which provide more details on constructs underlying poor adherence. Such recommendations stem from noted limitations of adherence measures relating to issues of sensitivity, specificity, and reproducibility. With pharmacy refill or claims data, participants listed limitations of lacking information on prescribing rationalization, being limited to selected populations (e.g. those with insurance coverage) or jurisdictions (e.g. those with resources and infrastructure), and failing to capture whether medications are actually taken. Other adherence measures discussed were pill counts and electronic monitoring, which were considered logistically prohibitive (e.g., costs, burden of sending to and retrieving from patients), and vulnerable to bias given that measures requiring patients’ involvement may serve as interventions themselves. Participants also discussed self-reported measures of adherence through interviews or questionnaires and noted that interviews were time-consuming, not always feasible in the clinic setting, and may overestimate adherence. Questionnaires on the other hand may not reflect actual medication taking behaviour, lack of validation, and have poor wording that may make it difficult to understand by patients. As participants indicated having “*no single perfect adherence measure*” using multiple measures would overcome specific limitations and allow triangulation of information captured by various sources.

#### c. Establishing clinically meaningful thresholds for adherence

Participants indicated the need to address the absence of a medication-specific and disease-specific clinically meaningful thresholds for “*good adherence*” in interventional studies of medication adherence. The conventional threshold of “*taking 80% of doses as prescribed*” is widely used to categorize patients as adherent or non-adherent and/or determine the effect of an intervention. A problem of using this threshold was that it did not distinguish between *“forgiving”* versus *“non-forgiving”* medications, that is, medications for which missing a small number of doses do not have significant clinical consequences versus one which can have severe consequences if not taken as prescribed. Consideration of clinically meaningful thresholds when designing adherence research studies was recommended instead of the current “*one-size-fits-all approach*” to better understand how much improvement in adherence is needed or necessary, how to translate improvements in adherence into clinical benefits, and whether the intervention is cost-effective.

### 2. Challenges in designing and appraising adherence intervention studies

This theme encapsulates key challenges with conducting adherence intervention studies as described from first-hand experiences by participants and extends to challenges with respect to evaluating/appraising adherence intervention studies.

#### a. Confusion over a plethora of outcomes when designing studies

An important challenge with conducting interventional studies of medication adherence identified by participants was the lack of a “*gold standard*” for outcomes leading to very little comparability between studies. Participants described outcomes they have measured and reported in their respective adherence intervention studies. “*Adherence outcomes*” included barriers to adherence (e.g. forgetfulness, ease of accessing the pharmacy), related constructs pertaining to medication taking (beliefs about medications, attitude to treatment, beliefs about adherence, and illness perception) and adherence itself (measured through varying methods such as self-report and pharmacy refill records). “*Clinical outcomes*” included condition-specific biological markers (e.g. serum uric acid level in gout), disease severity (e.g. disease activity, flare ups, pain), adverse events, and healthcare utilization (e.g. hospital visits, nurse appointments, primary care visits). Other outcomes described by participants represented psychological and psychosocial constructs including mental health (e.g. anxiety, depression), quality of life, and patient satisfaction. Finally, some participants reported measuring outcomes related to the intervention itself (e.g. cost, uptake, acceptability). Indeed, with such wide range of outcomes, participants indicated confusion on outcomes selection for intervention studies. Related to this, participants indicated the need to consider study participant burden and fatigue with respect to having too many outcomes to report.

#### b. Difficulties with powering studies to demonstrate meaningful changes

Another key challenge identified was study recruitment to achieve adequate statistical power. Participants highlighted this particular issue for several reasons. First, differences between intervention and comparison groups (effect sizes) are usually small, thus requiring bigger sample size (higher power) to be detected. Second, patients and funders were thought to be interested in changes in clinical outcomes rather than improvements in adherence, which may require an even bigger sample size, especially if power and sample size calculations are aimed at adherence as primary outcomes and clinical outcomes are considered as secondary outcomes. Third, individuals who participate in research studies are those who are more likely to be adherent, making it harder to detect additional effects of interventions. Indeed, non-adherent patients who are the population of interest for intervention studies and who may benefit the most were felt to be the hardest to recruit. Related to these issues of recruitment and sampling, participants indicated lack of consensus on whether sampling should be random or specifically targeted at non-adherent patients. In the case of targeted recruitment, participants were unsure of guidance on the best approach for screening potential participants and deciding who is non-adherent.

#### c. Suboptimal descriptions of adherence interventions in published studies

Participants indicated that challenges with adherence intervention studies are not confined to conducting studies themselves but also extend to evaluating/appraising published studies. A significant contributor to the latter challenge was poor reporting, that is description of adherence interventions, in published studies that limit the ability to understand their design. Participants indicated that very few authors describe how interventions have been conceptualized; for example, whether established frameworks (e.g. Health Belief Model, Necessity-Concerns Framework) were used [14, 15]. Participants also indicated that the lack of systematic use of terms that describe focus of the interventions (e.g. educational, behavioral, or affective) is a barrier in interpreting findings of adherence intervention studies.

### 3. Advancing outcome assessment in adherence intervention studies

This theme captures rationale for standardizing outcome assessment in adherence intervention studies and provided priorities and preferences for what outcomes should be included in a core domain set. It additionally captures challenges in developing and implementing a core domain set.

#### a. Rationale for a core domain set for adherence intervention studies

Participants noted the heterogeneity that has limited adherence intervention research and unanimously agreed on the importance of standardizing approaches to outcomes selection. According to participants, such standardization would make trials comparable, facilitate meta-analysis, allow combination of efforts internationally, and strengthen the evidence base (research and clinical knowledge) on what intervention works. With this, participants recognized the value of having a recommended core domain set for adherence intervention studies. Participants believed that a core domain set will improve not just interventional studies but all adherence research in general by adding rigor, offering clarity with regards to appropriate application of adherence measures, and promoting standardized use and reporting of outcomes. Having a core domain set was also believed to have positive impacts on clinical practice since it would inform clinicians on how best to support their patients with medication taking and reduce the many missed opportunities that currently exist in clinical practice due to inadequate evidence. Furthermore, participants indicated that a core domain set may improve quality of care at organizational levels by informing outcomes that should be measured in routine data collection for quality assurance purposes. One participant speculated that having a core domain set may actually reduce the number of outcomes that need to be measured to prove the effectiveness of an intervention, hence making the evaluation of interventions more feasible and “*cheaper*”.

#### b. Prioritizing outcomes for a core domain set for adherence intervention studies

Participants indicated a number of considerations for including outcomes in a core domain set, as well as shared preferences for types outcomes. The consideration of outcomes to be included in a domain set was believed to depend on: 1) the therapeutic area of focus (clinical outcomes in rheumatology vary significantly *between* rheumatic diseases and *from* other conditions, for example, cardiovascular disease); 2) culture of the target group (important when measuring the experience with the intervention); 3) research elements such as study objectives (pragmatic trials have different outcomes of interest than pilot studies) and study design (data source, data collection, analysis); 4) logistical factors including feasibility of implementing the outcome and funding availability (if researchers can afford to measure a certain outcome); and 5) intervention design (what it is designed to target, how frequently it is meant to be used). Finally, in no particular order, participants’ preferences for the types of outcomes in interventional studies targeting medication adherence included: 1) objective measures of adherence with attention to specific phases (e.g. initiation, implementation of the dosing regimen, persistence with therapy); 2) subjective measures of constructs that explain reasons for non-adherence (e.g. medication beliefs); 3) intervention-specific outcomes (outcomes that measure what the intervention was designed to target); 4) health outcomes which include composite, surrogate or direct clinical outcomes and side effects; 5) psychosocial outcomes that may be influenced by adherence (e.g. quality of life); and 6) economic outcomes (e.g. quality adjusted life years, cost, healthcare utilization, and medication wastage) that provide information on the cost-benefit of the intervention.

#### c. Challenges in developing and implementing a core domain set for adherence intervention studies

Along with providing rationale for a core domain set, participants also anticipated some challenges. Given complexities associated with aforementioned features and priorities for inclusion of outcomes in a core domain set (as described in the prior category), there were concerns around poor uptake. For example, the majority of participants were concerned that introduction of a core domain set might inhibit adherence research by recommending outcomes that may not be feasible or costly to measure. Some participants suggested that perhaps it would be better to provide guidelines around outcomes selection considerations as opposed to prescribing a list of outcomes to be measured. Some also believed that guidelines to standardize measurement methods might be more useful and practical than the recommendation of the core domain set since the same outcome can be measured using very different methods, providing different results. Participants questioned the appropriate extent to which the recommended core domain set should be imposed, that is, whether it should remain as a set of guidelines or be mandated under specific circumstances such as a requirement for funding or inclusion in a meta-analysis. Transparency in the benefits and consequences of following the recommendations was considered necessary to allow researchers to make an informed decision about whether or not to include the recommended outcomes in their study.

#### d. Inclusivity and representativeness of a core domain set for stakeholders in adherence intervention research

Participants highlighted that conducting adherence intervention studies often involves collaborations across diverse fields and interests – including patients and caregivers, psychologists, clinicians, regulators, clinical trialists, and epidemiologists. While lending strength to research, this was also noted as a challenge as each discipline has a different conceptual understanding of adherence. Participants anticipated that different groups will have different expectations from outcomes within a core domain set. It was, therefore, considered desirable for the core domain set to represent and reflect the priorities of different stakeholders. It was also recommended to provide a complementary glossary (terminology definition) with the recommended core domain set considering the diverse, heterogeneity and inconsistency of the terminology in adherence literature, and the significant disagreements between researchers in basic terminology and concepts in the field.

Participants felt that patients would be mostly interested in whether the intervention is going to help with their disease or interfere with their ability to undertake activities of daily living, quality of life (e.g. ability to sleep or have a social life. Participants indicated that researchers value biomarkers and surrogate markers of the disease in addition to aforementioned outcomes valued by patients and that clinicians would be interested in clinical outcomes as well as those that would inform them of the ease of implementing the intervention in practice. Finally, outcomes relevant to policy makers were considered to be utilization and cost-effectiveness of the intervention. Involvement of patients, clinicians and other stakeholders during the process of developing the core domain set was recommended to increase future uptake.

### Synthesis and resultant recommendations

In synthesizing findings across themes and categories, we identified researcher-informed recommendations for improving adherence research, which we summarize in Table 3. We also summarized researcher-informed recommendations for a core domain set for interventional studies targeting medication adherence, particularly in rheumatology, in Table 4.

**Table 3:** Researcher-informed recommendations for improving medication adherence research

Recommendation	CorrespondingTheme/Category
1. Specify the targeted adherence phase in designing interventions and studies	1a
2. Use multiple measures of adherence, considering *both* objective and subjective measures	1b
3. Use clinically meaningful thresholds for determining adherence/non-adherence(versus the current “one-size-fits-all” approach)	1c
4. Consider study participant burden and fatigue when determining number and types of outcomes	2a
5. Provide a glossary to define key terms when reporting adherence research studies	2c
6. Provide comprehensive descriptions of target, focus, and underlying conceptual framework(s),when designing and describing adherence interventions	2c
7. Consider outcomes that are:	3b
-accurately capture construct (e.g. adherence)	
-relevant (to the target patient population)	
-feasible (to implement)	
-valid (have sound measurement properties)	
-amenable to participants (measurement does not interfere with activities of daily living)

**Table 4:** Researcher-informed recommendations for developing a core domain set for interventional studies targeting medication adherence in rheumatology

Recommendation	CorrespondingTheme/Category
1. Specify benefits and consequences to support informed use of the core domain set byadherence researchers	3c
2. Involve patients, clinicians, decision makers, and other stakeholders in developing a coredomain set to represent and reflect respective priorities	3d
3. Consider the following aspects when establishing a core domain set	
-therapeutic area (e.g., clinical outcomes in rheumatology vary across conditions)	
- culture of the target group (particularly when the experience with the intervention)	
- research elements such as study objectives (pragmatic trials have different outcomes ofinterest than pilot studies) and study design (data source, data collection, analysis.	
-logistical factors including feasibility and funding availability (whether researchers can affordto measure a certain outcome)	
- the design of the intervention	
4. Prioritize the following features of outcomes in a core domain set:	3b
-relevance (to the target patient population)	
-feasibility (to implement, not cost-prohibitive)	
-validity (have sound measurement properties)	
5. Provide a complementary glossary for the core domain set to ensure consistent applicationand reporting	3d
6. Accompany the core domain set with guidelines to standardize measurement methods.	3d

## Discussion

This was an international qualitative study in which we interviewed 13 adherence researchers about their perspective in conducting adherence studies. Though we were particularly interested in *interventional* studies targeting adherence, participants’ sharing of their expertise and firsthand experiences with conducting intervention studies as well as other types of studies (e.g., descriptive studies on the burden of non-adherence, analytic studies to evaluate the impact of adherence on patient outcomes), enriched the interviews and insights gained. Altogether, thematic analysis led to the identification of three themes – improving measurement of adherence, challenges in designing and appraising adherence intervention studies, and advancing outcome assessment in adherence intervention studies. These themes have implications for informing recommendations for improving adherence research – including specifying the targeted adherence phase in designing adherence interventions and studies, using multiple measures of adherence, applying clinically meaningful thresholds for determining adherence/non-adherence, and providing a glossary to define key terms so as to continue promoting consistency in reporting of adherence research studies. Also, an implication of our study are identified researcher-recommendations for developing a core domain set for interventional studies targeting medication adherence including the involvement of patients, clinicians, decision makers and other stakeholders to represent respective priorities as well as methodological and practical considerations to establish rigor and support future uptake. While our findings have direct implications in rheumatology where we particularly focused our inquiry, we anticipate their applications in other chronic conditions.

Challenges in conducting and appraising adherence intervention studies were discussed at length by participants. This is not surprising considering that adherence research is a relatively new field, with the World Health Organization definition of adherence published less than two decades ago [16]. Our study confirms challenges in selecting an appropriate outcome in interventional studies targeting medication adherence and the ways in which the lack of guidance in this area hinders research and limits our ability to compare interventions and draw conclusions about their effectiveness. Many systematic reviews have noted the difficulty of combining results of adherence intervention studies because of the inconsistency in measurements in rheumatic and other chronic conditions [7, 17]. We found that the availability of a wide range of outcome domains and lack of recommendations or guidance contributes to this issue. This is reflected in a systematic review of the scope of outcomes in trials and observational studies of interventions targeting adherence in rheumatic conditions, where we identified 71 outcome domains in 53 studies [8]. Indeed, the researchers interviewed in our study shared having measured a wide variety of outcomes in the course of their career. Participants expressed preference for measures of objective and subjective adherence outcomes, health outcomes and intervention-specific outcomes, which is important to note because currently 23% of studies of adherence interventions in rheumatology have not reported the effect of their interventions on any health outcomes and only half reported medication adverse events [8].

An entire theme was identified around complexity of measuring adherence as an outcome for studies of interventions targeting medication adherence reflecting the multidimensional construct of adherence. The lack of a gold standard adherence measure and the many limitations of the current measures were particularly discussed. Our findings are in line with current knowledge about diversity of adherence measures and expands the findings of our aforementioned 2019 systematic review on the scope of outcomes in studies of adherence intervention in rheumatology, we found 115 unique measurements of adherence using 37 different instruments [8]. This international qualitative study adds to current literature by providing a robust and in-depth insight into how inconsistent measures of adherence results in poor assessment, understanding and comparison of adherence in practice [18–20]. Further, the perceptions and experiences shared by researchers interviewed in our study confirmed that the methodological shortcoming in measuring adherence in other therapeutic areas also apply to rheumatology including: lack of consideration of long-term trajectories of medication taking, lack of meaningful medication-specific and disease-specific adherence thresholds and poor distinction between the three phases of adherence [21–25].

Study findings have direct implications for informing our OMERACT Adherence Working Group’s efforts towards development of a core domain set for studies targeting adherence among patients with rheumatic diseases [9]. The complicated interaction of multiple factors that can impact the choice of outcomes for a certain study were discussed in depth by participants. Nonetheless, there was overall agreement among participants with respect to having a recommended domain set. Importantly, participants shared recommendations that span the development of the domain set as well as implementation and uptake.

Strengths of our study included collaborative development of the interview guide with Working Group members, interview pilot testing, purposive sampling for diversity of perspectives with member checking, investigator triangulation, and member checking. Limitations include interviewing only English speaking researchers and utilization of audio conferencing, which may have been associated with loss of non-verbal cues [26]. On balance, this set up was necessary to ensure feasibility given the goal of international recruitment of adherence researchers. Finally, our study was largely focused on adherence research among patients with rheumatic diseases. However, a number of researchers interviewed, particularly those who are non-clinicians in rheumatology, were also experienced in conducting adherence research in other fields. In addition, as many of the issues plaguing adherence research are not unique to rheumatology, our findings can apply to all studies of adherence interventions and potentially form the basis for recommendations for improving the design, conduct and evaluation across a wide spectrum of adherence research.

## Conclusion

Overall, adherence intervention research in rheumatology has been hindered by lack of standardization and guidance on terminology, measurement and outcome selection [7, 8]. Uniquely gathering perspectives of adherence researchers around the world, our study forms the basis for recommendations for improving the design, conduct and evaluation of adherence intervention studies in rheumatology, particularly for developing a core domain set of outcomes to improve consistency and facilitate comparisons. We also identified recommendations for developing a core domain set for interventional studies targeting medication adherence including involvement of patients, clinicians, and other stakeholders and methodological and practical considerations to establish rigor and support uptake.

## Supplementary Information

**Additional file 1.** Interview guide.

**Additional file 2.** The COREQ checklist.

## Data Availability

The datasets generated and/or analysed during the current study are not publicly available due the small sample size which makes it easy to identify the participants.

## Abbreviations

RA: Rheumatoid arthritis.
SLE: Systemic lupus erythematosus (SLE)
OMERACT: Outcome Measures in Rheumatology
