# Supplementary material for: Researchers’ perspectives on methodological challenges and outcomes selection in interventional studies targeting medication adherence in rheumatic diseases: an OMERACT-adherence study
Source: BMC Rheumatol. 2021 Jul 8;5:26. doi: 10.1186/s41927-021-00193-4 (PMC8265120; doi:10.1186/s41927-021-00193-4)
Supplement: Supplementary file 1 — Additional file 1. Interview guide. [file 41927_2021_193_MOESM1_ESM.docx]

# APPENDIX

**APPENDIX 1:** **Interview guide**

Thank you again for agreeing to participate in this interview. If it is okay with you, I would like to turn on the recorder, so we do not miss anything important.

Your study ID is ____ and the date is _____

This study entitled: Setting a core set of outcomes and recommendations for improving medication adherence intervention research in rheumatology (SHARE), involves interviewing experienced clinicians and researchers from around the world. Our aim is to describe clinician and researchers’ views on the design of studies that measure the impact of interventions aimed at improving patients’ adherence.

Any information you provide will be kept confidential. In order to maintain confidentiality during this interview, it can be helpful to use pseudonyms or pronouns when referring to yourself or others. We may use some direct quotations without identifying its author in the research publication. Please note that this interview will be audiotaped for research purposes only. This interview will take about half hour. Do you agree to continue?

Before I start with the interview, do you have any questions about the purpose of this research project?

**Introductory question**

- As a warm-up, could you please briefly describe the context of your adherence research in rheumatology?

1. **Challenges in adherence research design**
   1. Based on your experience, what are some key areas that challenging in designing and conducting adherence studies.
   2. Based on your experience, what are some key areas that challenging in evaluating findings of adherence studies.
2. **Outcomes currently measured and reported in adherence trials**
3. What adherence trials have you been involved in?
4. What were your primary and secondary outcomes?
5. How about clinical/psychosocial outcomes?
6. Based on your experience, what outcomes usually tend to be measured and reported, other than the ones you mentioned?
7. Which ones do you think best help us in understanding if our intervention works?
8. Can you identify any problems with the outcomes currently being reported? from patient/clinician/research perspective?
9. How do these problems impact the clinical decision-making and our understanding of the effectiveness of adherence interventions?
10. **Identifying important and relevant outcomes**
11. What outcomes (if you could suggest 1-3) do you believe to be relevant and important to include in all trials in testing adherence strategies and why? (impact on patients, clinical decision-making, burden on participants, economic evaluation, measures of disease activity, adverse events)
12. Do you think outcomes to be included depend on the context or design of the study?
13. **Core outcomes for rheumatology adherence studies**
14. There are increasing efforts to develop and test strategies to improve medication adherence in rheumatology, but comparison of these are hampered by differences in the design of these interventional studies, including outcome selection. Adherence studies to date have used heterogeneous adherence outcome measures, definitions and thresholds, and often have not assessed clinically meaningful health outcomes. Our group is trying to come up with a core domain set of outcomes. A core outcome set is an agreed minimum set of outcomes to be measured and reported in all adherence trials. What are your reactions and thoughts, in principle, about establishing core outcomes for trials testing strategies to improve adherence?
15. What are the potential benefits/pitfalls of recommending a core domain set of outcomes for ALL adherence intervention studies?
16. Can you suggest key criteria an outcome must fulfil to be included as a “core outcomes” –why? (feasibility, broadest impact, clinically relevant, cost)
17. What needs to be considered in implementing the core outcomes?
18. Do you think core outcomes could be used outside the trial context – why? (e.g. observational studies, registries, quality improvement)
19. What impacts (e.g. research, practice, policy, patient outcomes) do you think the implementation of core outcomes will have?
20. Do you have anything else you would like to share with me?
