# Supplementary material for: Researchers’ perspectives on methodological challenges and outcomes selection in interventional studies targeting medication adherence in rheumatic diseases: an OMERACT-adherence study
Source: BMC Rheumatol. 2021 Jul 8;5:26. doi: 10.1186/s41927-021-00193-4 (PMC8265120; doi:10.1186/s41927-021-00193-4)
Supplement: Supplementary file 2 — Additional file 2. The COREQ checklist. [file 41927_2021_193_MOESM2_ESM.docx]

|  |  | **Subtitle/**  **Page Number** | **Quote from Manuscript/explanation** |
| --- | --- | --- | --- |
| **Domain 1: Research team and reflexivity** | | | |
| **Personal Characteristics** | | | |
| 1. Interviewer/facilitator | Which author/s conducted the interview or focus group? | Data collection/  Page 5 | “The first author (SS), a PhD candidate, who is a pharmacist with a graduate degree in qualitative research, conducted the semi-structured interviews…” |
| 2. Credentials | What were the researcher’s credentials? E.g. PhD, MD | Data collection/  Page 5 | “The first author (SS), a PhD candidate, who is a pharmacist with a graduate degree in qualitative research, conducted the semi-structured interviews…” |
| 3. Occupation | What was their occupation at the time of the study? | NA | “The first author (SS), a PhD candidate, who is a pharmacist with a graduate degree in qualitative research, conducted the semi-structured interviews…” |
| 4. Gender | Was the researcher male or female? | NA | Researcher who conducted the interviews was female. |
| 5. Experience and training | What experience or training did the researcher have? | Data collection/  Page 5 | “The first author (SS), a PhD candidate, who is a pharmacist with a graduate degree in qualitative research, conducted the semi-structured interviews…” |
| **Relationship with participants** | | | |
| 6. Relationship established | Was a relationship established prior to study commencement? | NA | “Interviewer had no prior relationship with the participants” |
| 7. Participant knowledge of the interviewer | What did the participants know about the researcher? e.g. personal goals, reasons for doing the research | Appendix 1 |  |
| 8. Interviewer characteristics | What characteristics were reported about the interviewer/facilitator? e.g. Bias, assumptions, reasons and interests in the research topic | Data collection/  Page 5 | ““The first author (SS), a PhD candidate, who is a pharmacist with a graduate degree in qualitative research, conducted the semi-structured interviews…” |
| **Domain 2: study design Theoretical framework** | | | |
| 9. Methodological orientation and Theory | What methodological orientation was stated to underpin the study? e.g. grounded theory, discourse analysis, ethnography, phenomenology, content analysis | Analysis/Page 5 | “Data was analyzed using inductive thematic analysis following the steps recommended by Braun and colleagues” |
| **Participant selection** | | | |
| 10. Sampling | How were participants selected? e.g. purposive, convenience, consecutive, snowball | Participants/ page 5 | “We utilized purposive sampling to collect a broad range of perspectives and….” |
| 11. Method of approach | How were participants approached? e.g. face-to-face, telephone, mail, email | Analysis/ page 5 | “The first author…. conducted the semi-structured interviews through audio conference from August 2019 to January 2020” |
| 12. Sample size | How many participants were in the study? | Results/  page 7 | “Altogether, 13 (5 females) researchers…participated in the study” |
| 13. Non-participation | How many people refused to participate or dropped out? Reasons? | None | None |
| **Setting** | | | |
| 14. Setting of data collection | Where was the data collected? e.g. home, clinic, workplace | Analysis/ page 5 | “The first author…. conducted the semi-structured interviews through audio conference from August 2019 to January 2020” |
| 15. Presence of non-participants | Was anyone else present besides the participants and researchers? | Data collection/  Page 5 | The first author (SS… conducted the semi-structured interviews through audio conference from August 2019 to January 2020, without the presence of non-participants” |
| 16. Description of sample | What are the important characteristics of the sample? e.g. demographic data, date | Results/  page 7 | “Altogether, 13 researchers from seven countries (Australia, Belgium, Canada, Netherland, Thailand, United Kingdom, and United States of America) participated in the study ..all participants held a degree at least at the doctoral level and had led between two to five adherence research studies….majority worked in academia (75%) and specialized in epidemiology and/or health services research (61.5%) |
| **Data collection** | | | |
| 17. Interview guide | Were questions, prompts, guides provided by the authors? Was it pilot tested? | Analysis/ page 5 | “The interview guide (Appendix 1) was developed by Working Group members and pilot tested with one participant to determine question clarity, content validity, and average time required for completion” |
| 18. Repeat interviews | Were repeat interviews carried out? If yes, how many? | Analysis/ page 6 | “Results were shared with participants in a member-checking step to ensure accurate reflection of their shared perspectives” |
| 19. Audio/visual recording | Did the research use audio or visual recording to collect the data? | Analysis/ page 5 | “The first author (SS)…., conducted the semi-structured interviews through audio conference from August 2019 to January 2020” |
| 20. Field notes | Were field notes made during and/or after the interview or focus group? | Data collection/  Page 5 | “Field notes were taken as needed” |
| 21. Duration | What was the duration of the interviews or focus group? | Results/Page 7 | “Mean interview duration was 26 minutes.” |
| 22. Data saturation | Was data saturation discussed? | Analysis/Page 5 | “Saturation was defined as the point where no new insights on constructed themes emerged, as discussed and confirmed by the first and senior authors (SS, MDV)” |
| 23. Transcripts returned | Were transcripts returned to participants for comment and/or correction? | Analysis/ page 6 | “Results were shared with participants in a member-checking step to ensure accurate reflection of their shared perspectives” |
| **Domain 3: analysis and findings** | | | |
| **Data analysis** | | | |
| 24. Number of data coders | How many data coders coded the data? | Analysis/ page 5 | “The first author (SS) independently assigned as many different codes as relevant in a line-by-line approach using NVivo software” |
| 25. Description of the coding tree | Did authors provide a description of the coding tree? |  | N/A |
| 26. Derivation of themes | Were themes identified in advance or derived from the data? | Analysis/ page 5 | “Homogeneity and heterogeneity between the codes were then assessed to construct categories and eventual themes” |
| 27. Software | What software, if applicable, was used to manage the data? | Analysis/ page 5 | “The first author (SS) independently…. using NVivo software” |
| 28. Participant checking | Did participants provide feedback on the findings? Reporting | Analysis/ page 6 | “Results were shared with participants in a member-checking step to ensure accurate reflection of their shared perspectives” |
| 29. Quotations presented | Were participant quotations presented to illustrate the themes / findings? Was each quotation identified? e.g. participant number | Analysis/ page 6 | “Representative participant quotes were provided to illustrate the results.” |
| 30. Data and findings consistent | Was there consistency between the data presented and the findings? | Table 2 | “We describe each theme and corresponding categories in detail as follows and provide illustrative participant quotations in Table 2” |
| 31. Clarity of major themes | Were major themes clearly presented in the findings? | Results/page 7 | “We identified three themes: 1) improving measurement of adherence; 2) challenges in designing and appraising adherence intervention studies; and 3) advancing outcome assessment in adherence intervention studies” |
| 32. Clarity of minor themes | Is there a description of diverse cases or discussion of minor themes? | Discussion/  Pages 13 and 14 |  |
